# Draft Genome Sequences of Four Bacterial Species as Part of an Experiential Microbiology Project at SUNY Geneseo

**DOI:** 10.1128/MRA.01085-20

**Published:** 2020-11-19

**Authors:** Lauren N. Ellis, Enrico Amato, Christopher A. Lepore, Lucas B. Sutton, Bea Dipzinski, Brianna Cunneen, Julian Wisun, Kayla A. Cannon, Kyle O’Byrne, Isidro Bosch, Elizabeth Hutchison, Logan M. Peoples

**Affiliations:** a Department of Biology, SUNY Geneseo, Geneseo, New York, USA; University of Maryland School of Medicine

## Abstract

We report four draft genome sequences related to the genera *Bacillus* and *Escherichia*, recovered from surfaces associated with human interaction, and *Sediminibacterium*, recovered from an aquatic environment. This study was part of an undergraduate microbial bioinformatics course at the State University of New York at Geneseo.

## Abstract (precis)

Here, we report four draft genome sequences related to the genera *Bacillus* and *Escherichia*, recovered from surfaces associated with human interaction, and *Sediminibacterium*, recovered from an aquatic environment. This study was part of an undergraduate microbial bioinformatics course at the State University of New York at Geneseo.

## ANNOUNCEMENT

As part of a capstone project of an undergraduate bioinformatics course at the State University of New York (SUNY) at Geneseo, students sought to obtain genome sequences from microbes from human-associated environments. Students gained experience with key microbiological techniques consistent with proposed curricular guidelines (1), including microbial culturing, taxonomic identification, hypothesis-driven laboratory practices, and scientific communication. We report four draft genome sequences related to the genera *Bacillus*, *Escherichia*, and *Sediminibacterium*.

Samples were collected from the Integrated Science Center (ISC), located on the SUNY Geneseo campus, and Conesus Lake in Livingston County, New York, a source of drinking water and a recreational area prone to potentially toxic cyanobacterial blooms (2) (Table 1). Three samples were taken aseptically using sterile swabs from the following locations within the ISC: a computer keyboard in the chemistry stockroom, an elevator button, and a phone. The swabs were streaked onto Reasoner’s 2A agar and incubated at 37°C for 1 week. Colonies of interest were streaked onto tryptic soy agar and incubated at 37°C. After repeated subculturing, a single colony was inoculated into tryptic soy broth and incubated for 24 h at 37°C with shaking at 150 rpm to obtain sufficient biomass for DNA extraction.

**TABLE 1:** Identification and annotation information for each bacterial strain reported here

Isolate	Related strain (ANI [%])	Source	Size (Mbp)	Completeness (%)	Contamination (%)	Coverage (×)	No. of contigs	*N*_50_ value (bp)	GC content (%)	No. of genes	Genome accession no.	SRA accession no.
*Gen1*	Escherichia coli pK19EC149 (99.98)	Phone	4.54	99.96	0.08	73	87	110,270	50.72	4,378	JACBFE000000000	SRX8344491
*Gen2*	Bacillus licheniformis DSM 13 (99.99)	Elevator button	4.17	98.82	0.00	77	30	309,949	46.14	4,270	JACBFL000000000	SRX8344492
*Gen3*	Bacillus oleronius DSM 9356 (98.85)	Computer keyboard	5.45	98.30	2.04	77	59	183,139	34.99	5,471	JACBFM000000000	SRX8344493
*Gen4*	Sediminibacteirum goheungense DSM 28323 (75.82)	Conesus Lake enrichment	3.52	98.03	0.52	69	59	85,556	39.32	3,187	JACBFI000000000	SRX8344494

To isolate potential secondary metabolite-producing *Cyanobacteria*, freshwater was collected from Conesus Lake at a depth of 3 m using a Van Dorn discrete-depth sampling bottle during a picoplankton bloom in July 2019. To remove eukaryotic algae, a 100-ml subsample was serially filtered through 3.0-μm and 1.0-μm Whatman Nuclepore filters. The filtrate was inoculated into Alga-Gro freshwater medium (Carolina Biological Supply Company, Burlington, NC) and incubated at room temperature under a GrowLite fluorescent light (Barron Lighting Group, Glendale, AZ). The successful enrichment of *Cyanobacteria* was confirmed using epifluorescence microscopy. After repeated subculturing, a culture identified morphologically as *Synechococcus* was selected for whole-genome sequencing.

Genomic DNA was extracted from all cultures using a Mo Bio Ultraclean microbial DNA isolation kit (Qiagen, Hilden, Germany). Genomic DNA libraries were prepared using a Nextera XT kit (Illumina, San Diego, CA), and 150-bp paired-end reads were sequenced on a NextSeq 550 instrument at the Microbial Genome Sequencing Center (Pittsburgh, PA). Reads were quality trimmed using Trimmomatic (3) with the parameters LEADING:3 TRAILING:10 SLIDINGWINDOW:4:15. Paired-end reads of ≥100 bp were assembled using SPAdes v3.14.1 with the flag --careful (4). Contigs longer than 1 kb were retained. Genome statistics were analyzed using QUAST v5.0.2 (5). Completeness and contamination were determined using CheckM v1.0.13 with the --reduced_tree flag (6). Based on the CheckM output, we determined that the Conesus Lake cyanobacterial enrichment was not axenic and contained members related to *Synechococcus*, *Sediminibacterium*, and *Hydrogenophaga*. The contig depth of coverage was estimated using Bowtie 2 v2.4.1 and SAMtools v1.10 (7, 8), and contigs within this minimetagenome were binned using MetaBAT v0.26.3 with the flag --verysensitive (9). We report a genome bin obtained related to the genus *Sediminibacterium*. All four draft genomes were initially annotated using Prokka v1.14.6 (10), and final genome annotations are reported here using the NCBI Prokaryotic Genome Annotation Pipeline (11, 12). Average nucleotide identity (ANI) comparisons were performed using orthoANI (13).

While three of the genomes are closely related to previously reported strains, *Sediminibacterium* sp. strain Gen4 from Conesus Lake represents a novel species. We add to a growing body of evidence that members of the genus *Sediminibacterium* may be part of the cyanobacterial phycosphere (14, 15).

### Data availability.

This genome sequencing project has been deposited in GenBank under the accession number PRJNA631923.
